# JNJ-4178 (adafosbuvir, odalasvir, and simeprevir) in Japanese patients with chronic hepatitis C virus genotype 1 or 2 infection with or without compensated cirrhosis: the Phase IIa OMEGA-3 study

**DOI:** 10.1007/s00535-020-01672-0

**Published:** 2020-02-17

**Authors:** Tetsuo Takehara, Kazuaki Chayama, Masayuki Kurosaki, Hiroshi Yatsuhashi, Yasuhito Tanaka, Naoki Hiramatsu, Naoya Sakamoto, Yasuhiro Asahina, Akito Nozaki, Toshikazu Nakano, Yosuke Hagiwara, Hiroko Shimizu, Hiroki Yoshida, Yuhan Huang, Michael Biermer, Leen Vijgen, Norio Hayashi

**Affiliations:** 1 Department of Gastroenterology and Hepatology, Osaka University Graduate School of Medicine, 2-2 Yamada-oka, Suita, Osaka 565-0871 Japan; 2 Department of Gastroenterology and Metabolism, Graduate School of Biomedical and Health Sciences, Hiroshima University, 1-2-3, Kasumi, Minami-ku, Hiroshima, 734-8551 Japan; 3 Department of Gastroenterology and Hepatology, Musashino Red Cross Hospital, 1-26-1, Kyonan-cho, Musashino-shi, Tokyo, 180-8610 Japan; 4 Clinical Research Center, National Hospital Organization Nagasaki Medical Center, Kubara 2-1001-1, Omura, Nagasaki, 856-8562 Japan; 5 Department of Virology and Liver Unit, Nagoya City University Graduate School of Medical Sciences, Kawasumi, Mizuho, Nagoya, 467-8601 Japan; 6 Osaka Rosai Hospital, 1179-3 Kita, Sakai, Osaka 591-8025 Japan; 7 Department of Gastroenterology and Hepatology, Faculty of Medicine and Graduate School of Medicine, Hokkaido University, North 15 West 7 Kita-ku, Sapporo, Hokkaido 060-8638 Japan; 8 Department of Liver Disease Control, Department of Gastroenterology and Hepatology, Tokyo Medical and Dental University, 1-5-45 Yushima, Bunkyo-ku, Tokyo, 113-8519 Japan; 9 Gastroenterological Center, Yokohama City University Medical Center, 4-57 Urafune-cho, Minami-ku, Yokohama, 232-0024 Japan; 10 Janssen Pharmaceutical K.K, 5-2, Nishi-kanda 3-chome, Chiyoda-ku, Tokyo, 101-0065 Japan; 11 Clinical Pharmacology, Quantitative Sciences Division, R&D, Janssen Pharmaceutical K.K., 5-2, Nishi-kanda 3-chome, Chiyoda-ku, Tokyo, 101-0065 Japan; 12 Clinical Biostatistics Group 1 Biostatistics Department, 5-2, Nishi-kanda 3-chome, Chiyoda-ku, Tokyo, 101-0065 Japan; 13 Statistics and Decision Sciences, Janssen (China) Research and Development, LLC., 6F, Building A, Xinyan Mansion, No. 65 Guiqing Road, Xuhui District, Shanghai, People’s Republic of China; 14 Janssen Research and Development, Janssen Pharmaceutica NV, Turnhoutseweg 30, 2340 Beerse, Belgium; 15 Kansai Rosai Hospital, Inabasou 3-1-69, Amagasaki-shi, Hyogo, 660-8511 Japan

**Keywords:** AL-335, Hepatitis C virus, Japanese, Odalasvir, Simeprevir

## Abstract

**Background:**

The efficacy, safety, and pharmacokinetics of the combination of three direct-acting antiviral (DAA) agents (adafosbuvir [also known as AL-335], odalasvir, and simeprevir) were investigated in DAA treatment-naïve Japanese patients with genotype (GT)1 or GT2 chronic hepatitis C virus (HCV) infection, with or without compensated cirrhosis.

**Methods:**

In this Phase IIa, open-label, multicenter study—OMEGA-3 (NCT02993250)—patients received JNJ-4178 (adafosbuvir 800 mg once daily [QD], odalasvir 25 mg QD, and simeprevir 75 mg QD) for 8 (non-cirrhotic patients; Cohort 1) or 12 (cirrhotic patients; Cohort 2) weeks. Patients were followed-up to 24 weeks following the end of treatment (EOT). The primary endpoint was safety, including adverse events (AEs).

**Results:**

Overall, 33 patients were enrolled into Cohort 1 (*N* = 22) or 2 (*N* = 11) and received combined treatment with JNJ-4178. During the treatment and follow-up phases, a higher percentage of patients in Cohort 2 (81.8%) experienced AEs compared with Cohort 1 (68.2%), but the incidence of treatment-related AEs was similar. Most AEs were mild-to-moderate in severity and no patients discontinued due to an AE. There was one serious AE (cataract) in a patient in Cohort 2, which was not considered related to treatment. All patients achieved sustained virologic response 12 weeks after EOT (SVR12). No incidences of viral relapse were observed during follow-up.

**Conclusions:**

In HCV GT1- and GT2-infected Japanese patients, treatment with JNJ-4178 was well tolerated and resulted in 100% of patients achieving SVR12.

**Electronic supplementary material:**

The online version of this article (10.1007/s00535-020-01672-0) contains supplementary material, which is available to authorized users.

## Introduction

Japan has one of the highest rates of chronic hepatitis C virus (HCV) infection among the industrialized countries of the world [1]. Estimates of chronic HCV infection in Japan range from ~ 860,000 [1] to 2 million [2] cases, and HCV genotype (GT)1b and GT2a/b account for 65% and 34% of all cases, respectively [1]. Approximately 12% of HCV-infected patients in Japan have cirrhosis [2]. The 10-year survival rate for HCV-infected patients with cirrhosis in the absence of alcohol abuse has been estimated as 31–47% [3, 4], with most deaths attributable to hepatocellular carcinoma and hepatic failure [3]. This group of patients has been historically difficult to treat, with low sustained virologic response (SVR) rates achieved with traditional pegylated interferon (pegIFN) and ribavirin-based regimens [5].

The introduction of different classes of direct-acting antiviral (DAA) agents into clinical practice has significantly increased SVR rates among patients with HCV infection in recent years [6–8]. Current treatment guidelines for HCV infection recommend the use of IFN-free combinations of two or three DAAs [6–8]. Recommended treatment durations for DAA regimens are 8–16 weeks, depending on the HCV GT and the individual clinical characteristics of the patient [6–8]. Despite this, difficult-to-treat patients (including those with compensated cirrhosis) typically require longer treatment durations of up to 24 weeks [6–8]. Consequently, ongoing research efforts are centered on reducing treatment duration to achieve improved treatment adherence and tolerability, and reduced pill burden [9, 10].

Using a combination of DAAs with different mechanisms of action for the treatment of chronic HCV, it may be possible provide additive antiviral efficacy, thereby improving treatment outcomes, particularly for more difficult-to-treat patients (e.g., DAA-experienced patients and those with cirrhosis) [6–8, 11, 12]. Adafosbuvir, odalasvir, and simeprevir are three DAAs with distinct and different mechanisms of action.

Adafosbuvir (also known as AL-335) is a pro-drug of a uridine-based nucleotide analog polymerase (NS5B) inhibitor with potent antiviral activity against HCV GTs 1–6 [13–15].

Odalasvir is an investigational HCV NS5A inhibitor with picomolar potency [16] against HCV GT1, GT2, GT4, GT5, and GT6 [17], and nanomolar activity against HCV GT3 (data on file) [16].

Simeprevir is a HCV NS3/4A protease inhibitor with demonstrated antiviral activity in GT1-, GT2-, GT4-, GT5-, and GT6-infected patients, but no antiviral activity in GT3-infected patients [18]. Simeprevir is metabolized by cytochrome P450 (CYP) 3A. It is also a mild inhibitor of intestinal (not hepatic) CYP3A and a mild, but not clinically relevant, inhibitor of CYP1A2 [19]. Simeprevir 100 mg was approved for use in combination with pegIFN and ribavirin in patients with HCV GT1 infection without cirrhosis in Japan [20], before simeprevir was withdrawn from the market at the end of March 2019. Simeprevir 150 mg was also approved for use in patients with HCV GT1 or GT4 infection, with and without compensated cirrhosis, in North America and Europe [21, 22]. In addition, simeprevir was approved for use as part of an IFN-free combination with sofosbuvir for the treatment of patients with HCV GT1 infection in North America, and for HCV GT1 and GT4 infection in Europe [21, 22]. The withdrawal of simeprevir came after the sponsor’s global decision to discontinue further development of the simeprevir-containing 3-DAA treatment regimen studied here—a decision that was not driven by any safety or efficacy concerns [23].

The 3-DAA combination of adafosbuvir, odalasvir, and simeprevir was evaluated in two Phase II studies: a Phase IIa study (AL-335-604; NCT02569710) in HCV-infected patients with compensated cirrhosis (data on file) or without compensated cirrhosis [14], and the Phase IIb OMEGA-1 study (NCT02765490) of HCV GT1-, GT2-, GT4-, and GT5-infected patients without cirrhosis [16]. In the AL-335-604 study, SVR rates 12 and 24 weeks after the end of treatment (EOT; SVR12 and SVR24, respectively) of 100% were achieved in 40 GT1-infected patients without cirrhosis following 6 or 8 weeks’ treatment with adafosbuvir 800 mg once daily (QD), odalasvir 50 mg every other day, and simeprevir 75 mg QD, and the regimen was generally well tolerated [14]. The results of the AL-335-604 study were used to determine the dose of each component in the 3-DAA combination JNJ-4178—adafosbuvir 800 mg QD, odalasvir 25 mg QD, and simeprevir 75 mg QD—which was evaluated in the OMEGA-1 study. In OMEGA-1, 365 patients without cirrhosis received JNJ-4178 combined treatment for 6 or 8 weeks and achieved SVR12 rates of 98.9% and 97.8%, respectively. Notably, SVR12 rates > 99% were reported for HCV GT1-, GT4-, and GT5-infected patients. JNJ-4178 was well tolerated, with most adverse events (AEs) mild in severity and no evidence of cardiac toxicity on detailed cardiac evaluation [16].

Here, we describe the results of the Phase IIa OMEGA-3 study (NCT02993250), which evaluated the safety, efficacy, and pharmacokinetics of 8 and 12 weeks of JNJ-4178 treatment in Japanese HCV GT1- or GT2-infected patients, with or without compensated cirrhosis. Dosing was guided by the results of the Phase IIa AL-335-604 study, as well as data from Phase I studies, such as the AL-335-602 study (NCT02512562) [24], and study HPC1006 (NCT02824315) in healthy Japanese volunteers (data on file).

## Methods

### Study design

**Fig. 1:**
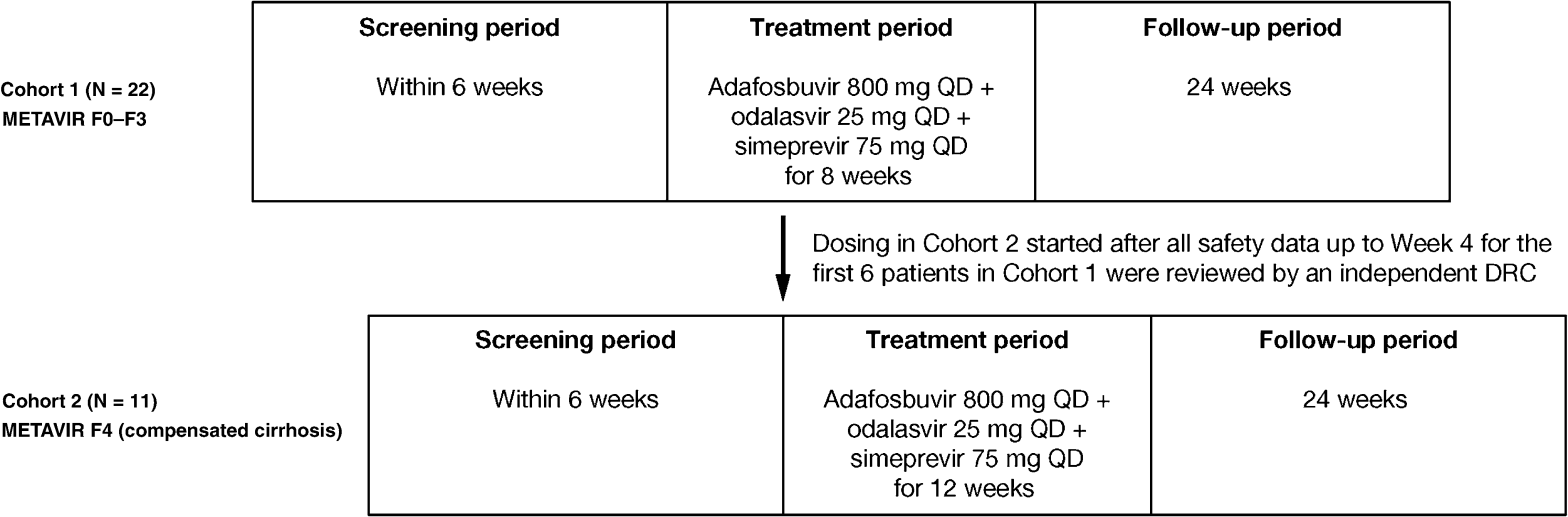
OMEGA-3 study design. *DRC* data review committee, *QD* once daily

OMEGA-3 was a Phase IIa, multicenter, open-label study conducted at 16 sites in Japan between December 22, 2016 and May 7, 2018. The study comprised a 6-week screening phase, an 8-week (Cohort 1: patients with no cirrhosis) or 12-week (Cohort 2: patients with compensated cirrhosis) open-label treatment phase, and a 24-week post-treatment follow-up period. Given that patients with HCV and compensated cirrhosis potentially have a higher safety risk than patients without compensated cirrhosis, dosing of Cohort 2 was only initiated after all safety data had been reviewed up to Week 4 for the first six patients in Cohort 1 by an independent Data Review Committee (Fig. 1). This study was conducted in accordance with the ethical principles that have their origin in the Declaration of Helsinki and that are consistent with Good Clinical Practices and applicable regulatory requirements. The study was approved by the relevant Institutional Review Board at each study center. All patients provided written, informed consent to participate.

### Patients

Eligible patients were aged 20–75 years, with a body mass index of 18–35.0 kg/m^2^, and documented HCV GT1 or GT2 infection for at least 6 months, with or without compensated cirrhosis. Presence of cirrhosis was defined as a FibroScan result of > 12.5 kPA [25] or liver biopsy showing the presence of cirrhosis (METAVIR score of F4 or Ishak score of ≥ 5), either as a prior report or during the screening phase. Patients were also required to have a plasma HCV ribonucleic acid (RNA) level of ≥ 10,000 IU/mL at screening and to be DAA treatment naïve (no prior exposure to any approved or investigational DAAs). Prior HCV therapy comprising IFN (pegylated or non-pegylated), with or without ribavirin, was permitted.

Key exclusion criteria included HCV GT3, GT4, GT5, or GT6 infection, co-infection with multiple HCV GTs, human immunodeficiency virus or hepatitis B virus, any liver disease of non-HCV etiology, evidence or history of hepatic decompensation as assessed with Child–Pugh Class B or C (history or current clinical evidence of ascites, bleeding varices, or hepatic encephalopathy), history or evidence of significant cardiac findings (including evidence of cardiac disease, screening echocardiogram left ventricular ejection fraction [LVEF] < 55% or any other finding suggestive of clinically relevant cardiomyopathy, abnormal electrocardiogram [ECG] findings, heart block, and history or family history of prolonged QT syndrome or sudden cardiac death), or pre-specified laboratory abnormalities at screening (platelet count < 75 × 10^9^/L; hemoglobin < 110 g/L for male patients and < 100 g/L for female patients; absolute neutrophil count < 1.00 × 10^9^/L; aspartate aminotransferase [AST] or alanine aminotransferase [ALT] > 10 × upper limit of normal [ULN]; total serum bilirubin > 1.5 × ULN; albumin < 35 g/L; estimated glomerular filtration rate of < 50 mL/min/1.73 m^2^ and hypo-/hyperkalemia ≥ Grade 2).

Concomitant use of the following drugs was not permitted: potent and moderate CYP3A4 inducers, and P-glycoprotein inhibitors from 2 weeks before baseline until EOT; potent and moderate CYP3A4 inhibitors, and CYP3A substrates with a narrow therapeutic index, from baseline until EOT; drugs associated with QT prolongation and/or torsades de pointes from screening until end of the study; immunomodulators, proton pump inhibitors, anti-arrhythmics, beta-blockers, calcium/sodium/potassium channel blockers, and herbal products for HCV treatment from screening until EOT; drugs for improvement of hepatic function from screening until Week 12 of follow-up; and oral contraceptives from 2 weeks before baseline until Week 4 of follow-up. Consumption of large quantities of grapefruit juice (> 1 L/day) was also prohibited throughout the study from baseline until EOT.

Patients received JNJ-4178 (adafosbuvir 800 mg QD, odalasvir 25 mg QD, and simeprevir 75 mg QD) for either 8 (Cohort 1: patients with no cirrhosis) or 12 weeks (Cohort 2: patients with compensated cirrhosis). Study drugs were administered orally with food at approximately the same time in the morning, each day.

### Study endpoints

The primary endpoint was safety, including but not limited to AEs, physical examination, vital signs, 12-lead ECGs, echocardiograms, and clinical laboratory results.

Secondary endpoints included the proportion of patients with SVR12 and SVR24, the proportion of patients with on-treatment virologic response (HCV RNA not detected or HCV RNA < lower limit of quantification [LLOQ]), time to achieve HCV RNA not detected or HCV RNA < LLOQ, and the proportion of patients with viral relapse (no SVR12 with HCV RNA < LLOQ at EOT and confirmed HCV RNA ≥ LLOQ during follow-up) or on-treatment failure (no SVR12 with confirmed HCV RNA ≥ LLOQ at EOT), and the plasma pharmacokinetics of adafosbuvir (and its metabolites ALS-022399 [monophosphate precursor] and ALS-022227 [parent nucleotide]), odalasvir, and simeprevir.

The effect of the presence or absence at baseline of HCV NS5A, NS5B, and/or NS3/4A polymorphisms on treatment outcome was included as an exploratory endpoint.

### Study assessments

#### Safety

AEs were reported throughout the study from the time that informed consent to participate was received until completion of the last visit. Complete physical examination, vital signs assessments, and blood and urine sampling for clinical laboratory assessments (including hematological, serum chemistry, liver function, and urinalysis tests) were performed at screening and throughout the study. Triplicate ECGs and echocardiography were also performed at screening and during the study.

#### Efficacy

HCV RNA was measured quantitatively at screening, baseline (pre-dose), Days 2 and 3, Weeks 1, 2, 3, 4, 6, 8, and 10 (Cohort 2 only), EOT (Weeks 8 or 12), and follow-up (Weeks 4, 8, 12, 18, and 24). Blood samples were processed centrally in real time using an in vitro nucleic acid amplification test (Cobas^®^ AmpliPrep/Cobas^®^ TaqMan^®^ HCV Test v2.0, Roche Molecular Diagnostics) with an LLOQ and limit of detection of 15 IU/mL.

Samples for sequencing of the HCV NS3/4A, NS5A, and NS5B regions were collected at baseline (pre-treatment) and at the same time points as noted above for HCV RNA, with the exception of Days 2 and 3. The HCV NS3/4A, NS5A, and NS5B regions were planned to be sequenced post-baseline in patients not achieving SVR using next-generation sequencing (NGS; Illumina, San Diego, CA, USA) with a 1% read frequency cut-off.

For NS5A and NS5B, the resistance analyses considered lists of amino acid positions of interest that are associated with resistance to the class of NS5A or nucleotide analog NS5B inhibitors, including positions of specific interest for odalasvir or adafosbuvir based on in vitro or clinical observations [13, 26, 27]. For NS3, the analyses focused on known simeprevir resistance-associated substitutions (RASs, substitutions with an in vitro fold change in 50% effective concentration of simeprevir > 2) [13, 21, 22, 28]. Amino acid substitutions were defined as changes from reference sequence: GT1a H77 (GenBank Accession ID NC_004102), GT1b Con1 (AJ238799), and GT2a JFH1 (AB047639) for GT1a, GT1b, and GT2 samples, respectively. Baseline polymorphisms were defined as amino acid substitutions detected at baseline with an NGS read frequency of ≥ 15%.

At the baseline visit, a blood sample to determine host *IL28B* genotype (rs12979860) was collected.

#### Pharmacokinetics

Blood samples for the assessment of the plasma pharmacokinetics of adafosbuvir (and its metabolites ALS-022399 and ALS-022227), odalasvir, and simeprevir were collected in all patients pre-dose, between 2 and 4 h post-dose, and between 4 and 6 h post-dose at Weeks 2, 4, 6, and 8 (Cohort 2 only) and EOT (Weeks 8 or 12). An additional plasma sample was taken at any time during the Week-12 follow-up visit for measurement of odalasvir only.

Additional blood samples were also obtained at Week 4 in a subgroup of patients (planned: ≥ 6 patients per cohort) who consented to intensive pharmacokinetic sampling (pharmacokinetic sub-study). Blood samples for adafosbuvir (and its metabolites ALS-022399 and ALS-022227), odalasvir, and simeprevir plasma concentrations were collected pre-dose and at 1, 2, 3, 4, 6, 8, 10, 12, and 24 h post-dose.

Plasma concentrations of adafosbuvir, ALS-022399, ALS-022227, simeprevir, and odalasvir were determined using validated liquid chromatography–mass spectrometry methods. The LLOQ for adafosbuvir and odalasvir was 1.00 ng/mL, for ALS-022399 was 2.00 ng/mL, for ALS-022227 was 5.00 ng/mL, and for simeprevir was 20.0 ng/mL.

### Statistical analysis

As this was an exploratory study, no formal sample size calculation was performed. A total sample size of approximately 40 patients (20 per cohort) was planned to explore the safety and efficacy of JNJ-4178 in the study population.

The safety analysis was performed on the Safety Set (all patients who received at least one dose of study drug). Safety data were analyzed descriptively. AEs were coded using the Medical Dictionary for Regulatory Activities (version 20.0). Laboratory abnormalities were assessed according to the World Health Organization Toxicity Grading Scale.

The efficacy analysis was performed on the Full Analysis Set (all patients who received at least one dose of study drug [adafosbuvir, odalasvir, or simeprevir] and had at least one post-baseline efficacy assessment). For each treatment cohort, the proportions of patients who achieved SVR12 and SVR24, along with two-sided 95% confidence intervals (CIs), were calculated. Descriptive statistics were used for all efficacy endpoints.

Descriptive statistics for plasma concentrations of adafosbuvir (and its metabolites), odalasvir, and simeprevir were calculated at each sampling time point. Pharmacokinetic parameters were derived by non-compartmental pharmacokinetic analysis of adafosbuvir (and its metabolites), odalasvir, and simeprevir in the pharmacokinetic sub-study using actual sampling time points and plasma concentrations obtained during intensive sampling at Week 4. Graphs of the mean plasma concentration–time profiles were produced. An exploratory graphical pharmacokinetic/pharmacodynamic analysis was performed by means of scatter plots of pharmacodynamic parameters (individual PR interval [mean of the triplicate ECG measurements] and change from baseline in PR interval [Day 1 was baseline]) versus odalasvir plasma concentrations.

## Results

### Patients

**Fig. 2:**
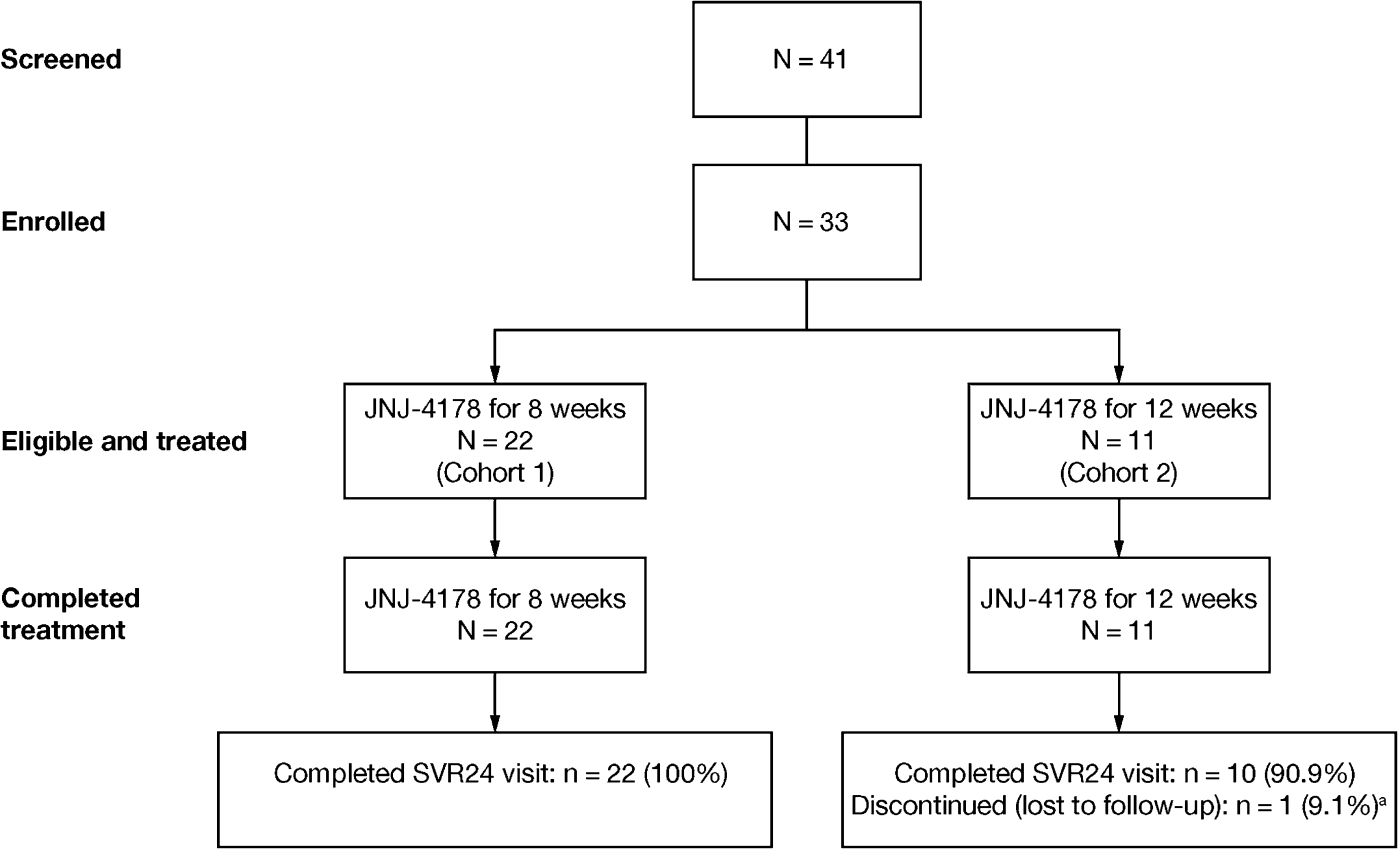
Patient disposition. ^a^One patient in Cohort 2 discontinued the study due to non-clinical reasons, after having achieved SVR12. *JNJ-4178* adafosbuvir 800 mg QD + odalasvir 25 mg QD + simeprevir 75 mg QD, *QD* once daily, *SVR12/24 *sustained virologic response 12/24 weeks after end of treatment

A total of 33 patients were enrolled and received either 8 weeks’ (Cohort 1: patients without cirrhosis, *N* = 22) or 12 weeks’ (Cohort 2: patients with compensated cirrhosis, *N* = 11) treatment (Fig. 2). Due to the global decision to discontinue further development of JNJ-4178 (adafosbuvir, odalasvir, and simeprevir) for the treatment of chronic HCV infection, enrollment into OMEGA-3 was completed prior to reaching the target of 20 patients with cirrhosis in Cohort 2 [23]. All patients completed treatment. One patient in Cohort 2 discontinued the study after the Week-12 follow-up visit for non-clinical reasons (lost to follow-up).

Baseline characteristics and demographics of the two patient cohorts are presented in Table 1. A greater proportion of patients in Cohort 1 were female compared with those in Cohort 2 (77.3% [17/22] vs 36.4% [4/11]). The majority of patients in both cohorts were infected with HCV GT1b (Cohort 1: 68.2% [15/22]; Cohort 2: 72.7% [8/11]). All other patients were infected with HCV GT2 (Cohort 1: 31.8% [7/22], Cohort 2: 27.3% [3/11]). Most (81.8% [18/22]) of the patients in Cohort 1 were categorized as METAVIR fibrosis stage F0–F2. As per protocol, all patients in Cohort 2 had compensated cirrhosis. Most patients (90.9% [10/11]) in Cohort 2 were categorized as METAVIR fibrosis stage F4 (determined by FibroScan in nine patients, and by liver biopsy in one patient) and one patient (9.1%) was categorized as Ishak fibrosis stage five (determined by liver biopsy).

**Table 1:** Patient baseline demographic and clinical characteristics

Characteristic^a^	Cohort 1: patients with no cirrhosis (*N* = 22)	Cohort 2: patients with compensated cirrhosis (*N* = 11)	Total (*N* = 33)
Female, *n* (%)	17 (77.3)	4 (36.4)	21 (63.6)
Age, years	59 (27–74)	55 (42–67)	58 (27–74)
BMI, kg/m^2^	23.3 (18.2–33.5)	23.6 (18.4–29.6)	23.4 (18.2–33.5)
Baseline HCV RNA, log_10_ IU/mL	6.23 (3.82–6.96)	5.86 (4.67–6.69)	6.11 (3.82–6.96)
Baseline HCV RNA ≥ 6,000,000 IU/mL, *n* (%)	5 (22.7)	0	5 (15.2)
HCV geno/subtype, *n* (%)			
1b	15 (68.2)	8 (72.7)	23 (69.7)
2a	6 (27.3)	2 (18.2)	8 (24.2)
2b	1 (4.5)	1 (9.1)	2 (6.1)
METAVIR fibrosis score,^b^*n* (%)			
F0−F2	18 (81.8)		18 (54.5)
F3	4 (18.2)	0	4 (12.1)
F4	0	10 (90.9)	10 (30.3)
Ishak fibrosis stage 5^c^	0	1 (9.1)	1 (3.0)
FibroScan score, kPa	6.8 (3.3–12.0)	20.2 (13.1–34.3)	11.5 (3.3–34.3)

*BMI* body mass index, *HCV* hepatitis C virus, *IL28B* interleukin 28B, *RNA* ribonucleic acid

^a^Mean (range) values are presented unless otherwise indicated. ^b^Based on FibroScan or liver biopsy

^c^Based on liver biopsy

Baseline polymorphisms considering the NS3, NS5A, and NS5B positions of interest by baseline genotype are shown in Supplementary Table S1. Baseline polymorphisms considering NS5A positions 28, 29, 30, 31, 32, 58, 92, and 93 were present in 10/23 (43.5%) GT1b- and 10/10 (100.0%) GT2-infected patients. None of the GT1b-infected patients had NS3 simeprevir RASs, while all 10 GT2-infected patients had a baseline NS3 simeprevir RAS I132L, which was present in combination with simeprevir RAS K122R in 3 of these patients. The NS5B S282T substitution, associated with in vitro resistance to adafosbuvir, was not detected in any patients at baseline.

### Safety

During the treatment and follow-up phase, 72.7% of all patients reported at least one AE (Cohort 1: *n* = 15 [68.2%], Cohort 2: *n* = 9 [81.8%]; Table 2). No patient discontinued treatment or withdrew consent due to an AE. There was one serious AE (aggravated cataract requiring hospitalization) in a patient in Cohort 2, which was not considered related to treatment. The most common AEs, occurring in > 10% of patients overall, were viral upper respiratory tract infection (Cohort 1: *n* = 1 [4.5%], Cohort 2: *n* = 4 [36.4%]), headache (Cohort 1: *n* = 3 [13.6%], Cohort 2: *n* = 2 [18.2%]), and increased ALT or AST (Cohort 1: *n* = 2 [9.1%], Cohort 2: *n* = 2 [18.2%], for each of these AEs). Most AEs were mild-to-moderate in severity. During the treatment phase, 9.1% of all patients (Cohort 1: *n* = 2, Cohort 2: *n* = 1) experienced Grade 3 (severe) or 4 (potentially life-threatening) AEs. Grade 3 AEs included increased AST (Cohort 1: *n* = 1), increased amylase (Cohort 1: *n* = 1), and cataract (Cohort 2: *n* = 1). No relationship between increased amylase or cataract and study treatment was identified. The patient with Grade 3 increased AST also experienced Grade 4 increased ALT, both occurred on Day 56 and were considered possibly related to treatment. The patient’s HCV RNA levels were negative during this flare and, as the treatment period had already been completed at notification, the study drugs were not discontinued due to this AE, which was considered as resolved on Day 84.

**Table 2:** Summary of adverse events during treatment and follow-up

*n* (%)	Cohort 1: patients with no cirrhosis (*N* = 22)	Cohort 2: patients with compensated cirrhosis (*N* = 11)	Overall (*N* = 33)
Any AE	15 (68.2)	9 (81.8)	24 (72.7)
Worst Grade 1–2	13 (59.1)	8 (72.7)	21 (63.6)
Worst Grade 3	1 (4.5)	1 (9.1)	2 (6.1)
Worst Grade 4	1 (4.5)	0	1 (3.0)
Treatment-related AE (any drug)	9 (40.9)	4 (36.4)	13 (39.4)
At least possibly related to adafosbuvir	6 (27.3)	3 (27.3)	9 (27.3)
At least possibly related to odalasvir	9 (40.9)	4 (36.4)	13 (39.4)
At least possibly related to simeprevir	6 (27.3)	2 (18.2)	8 (24.2)
Any AE with a fatal outcome	0	0	0
Any SAE	0	1 (9.1)	1 (3.0)
At least possibly related to any study drug	0	0	0
AE leading to permanent discontinuation^a^	0	0	0
Most common AEs^b^			
Viral upper respiratory tract infection	1 (4.5)	4 (36.4)	5 (15.2)
Headache	3 (13.6)	2 (18.2)	5 (15.2)
Alanine aminotransferase increased	2 (9.1)	2 (18.2)	4 (12.1)
Aspartate aminotransferase increased	2 (9.1)	2 (18.2)	4 (12.1)
Diarrhea	3 (13.6)	0	3 (9.1)
First-degree atrioventricular block	2 (9.1)	1 (9.1)	3 (9.1)
Abdominal pain upper	2 (9.1)	0	2 (6.1)
Gastritis	0	2 (18.2)	2 (6.1)
Neck pain	1 (4.5)	1 (9.1)	2 (6.1)
Malaise	2 (9.1)	0	2 (6.1)

*AE* adverse event, *SAE* serious adverse event

^a^Permanent discontinuation of at least one study drug. ^b^≥ 5% of patients in the total group

The following events of interest were reported in Cohorts 1 and 2, respectively: cardiac events, *n* = 5 (22.7%) and *n* = 2 (18.2%); and rash, *n* = 1 (4.5%) and *n* = 0. All AEs of interest were Grade 1 or 2. Two patients (9.1%) in Cohort 1 and one patient (9.1%) in Cohort 2 experienced first-degree atrioventricular block; all events were considered either possibly or probably related to the study drugs. Other cardiac events included supraventricular extra systoles in one patient in Cohort 2 (considered doubtfully related to the study drugs), increased blood creatine phosphokinase and ECG QT prolongation in one patient each, both in Cohort 1 (considered possibly related to any one of the study drugs), and brain natriuretic peptide levels above normal (105 ng/L [upper limit of normal: 100 ng/L]) in one patient in Cohort 1 (considered possibly related to odalasvir). There were no incidences of increased bilirubin, photosensitivity conditions, or pruritus reported as AEs.

During the treatment or follow-up periods, four patients (18.2%) in Cohort 1 and one patient in Cohort 2 (9.1%) experienced treatment-emergent QTcF interval prolongation > 450 ms, but no patient experienced prolongation > 480 ms. Two patients (9.1%) in Cohort 1 and 1 patient (9.1%) in Cohort 2 had heart rates ≤ 50 bpm. There were no QRS abnormalities > 120 ms. Increases in PR interval > 200 ms were observed in two patients (9.1%) in Cohort 1 and 1 patient (9.1%) in Cohort 2. All PR interval increases were Grade 1 (0.20–0.25 s) or 2 (> 25 s). A decline in LVEF of > 5% to ≤ 10% was observed in 3 patients (13.6%) in Cohort 1 and 1 patient (9.1%) in Cohort 2. However, an increase in LVEF of > 5% to ≤ 10% was also observed in 3 patients (Cohort 1: *n* = 1 [4.5%]; Cohort 2: *n* = 2 [18.2%]). None of the echocardiogram abnormalities were considered to be clinically significant.

Other than the cases of increased AST, ALT, and amylase that were recorded as AEs and are described above, all laboratory abnormalities were Grade 1 or 2.

### Efficacy

SVR12 rates (95% CI) were 100% (84.6–100%) in Cohort 1 (22/22) and 100% (71.5–100%) in Cohort 2 (11/11) (Table 3). SVR24 rates (95% CI) were 100% (84.6–100%) in Cohort 1 and 90.9% (58.7%–99.8%) in Cohort 2. One patient in Cohort 2 was lost to follow-up after having achieved SVR12. No patients experienced on-treatment failure or viral relapse.

**Table 3:** Virologic response during and after treatment, including viral relapse (Full Analysis Set)

*n*/*N* (%)	Cohort 1: patients with no cirrhosis (*N* = 22)	Cohort 2: patients with compensated cirrhosis (*N* = 11)
During treatment		
Week 2		
HCV RNA not detected	5 (22.7)	5 (45.5)
HCV RNA < LLOQ^a^	11 (50.0)	7 (63.6)
Week 4		
HCV RNA not detected	16 (72.7)	8 (72.7)
HCV RNA < LLOQ^a^	21 (95.5)	9 (81.8)
End of treatment^b^		
Week 8		
HCV RNA not detected	22 (100)	11 (100)
HCV RNA < LLOQ^a^	22 (100)	11 (100)
Week 12		
HCV RNA not detected	NA	11 (100)
HCV RNA < LLOQ^a^	NA	11 (100)
After treatment		
SVR12 [95% CI]	22/22 (100) [84.6–100)	11/11 (100) [71.5–100]
SVR24 [95% CI]	22/22 (100) [84.6–100]	10/11 (90.9) [58.7–99.8]

*CI* confidence interval, *GT* genotype, *HCV* hepatitis C virus, *LLOQ* lower limit of quantification, *NA* not applicable, *RNA *ribonucleic acid, *RT-qPCR* real-time reverse transcription quantitative polymerase chain reaction, *SVR12/24* sustained virologic response 12 or 24 weeks after the end of treatment

^a^The LLOQ of the HCV RT-qPCR was 15 IU/mL

^b^End of treatment was Week 8 for Cohort 1 and Week 12 for Cohort 2. Week 8 values for Cohort 2 are provided for completeness

**Fig. 3:**
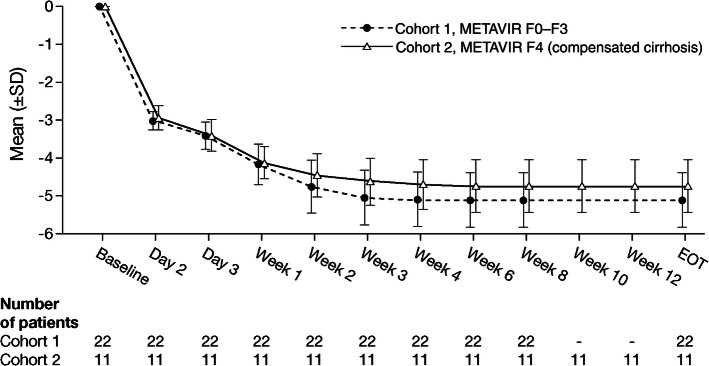
On-treatment mean change from baseline in HCV RNA (log_10_ IU/mL) (Full Analysis Set). HCV RNA values were set to 14 when values were < 15 IU/mL detected, and to 13 when values were < 15 IU/mL not detected. *EOT* end of treatment, *HCV* hepatitis C virus, *RNA* ribonucleic acid, *SD* standard deviation

The proportion of patients with on-treatment virologic response (HCV RNA not detected or HCV RNA < LLOQ, detected or not detected) increased with duration of treatment (Table 3; Fig. 3). By Week 2, 50% and 63.6% of patients had HCV RNA < LLOQ and 22.7% and 45.5% had HCV RNA not detected in Cohorts 1 and 2, respectively. By Week 8, all patients in both cohorts had HCV RNA not detected.

Mean (standard deviation [SD]) time to on-treatment virologic response was 19.0 (1.68) days for Cohort 1 and 18.6 (3.86) days for Cohort 2.

### Pharmacokinetics

Adafosbuvir and ALS-022399 plasma trough concentration (*C*_trough_) levels were below the limit of quantification at all visits in both patient cohorts. ALS-022227 *C*_trough_ levels were similar at all visits, suggesting that steady-state exposure had been reached by Week 2. Odalasvir *C*_trough_ levels increased modestly during treatment at Weeks 2, 4, 6, and 8, indicating that steady-state conditions had not entirely been reached yet at Week 4. In single-dose pharmacokinetic studies (data on file), the terminal elimination half-life was approximately 250 h, and likely contributed to this finding [24]. Simeprevir *C*_trough_ levels were relatively similar between visits, suggesting that steady-state exposure had been reached by Week 2; however, between-patient variability in *C*_trough_ levels was high (min 92.2 ng/mL; max 6090 ng/mL). *C*_trough_ levels were similar between the cohorts for ALS-022227, modestly lower in Cohort 2 compared with Cohort 1 for odalasvir, and higher in Cohort 2 compared with Cohort 1 for simeprevir.

Mean maximum observed analyte concentration (*C*_max_) and area under the plasma concentration–time curve 0–24 h post-dose (AUC_24h_) in the pharmacokinetic sub-study conducted at Week 4 are presented in Supplementary Table S2. As only two patients in Cohort 2 participated in the intensive pharmacokinetic sub-study, due to the early termination of enrollment in this cohort, any comparison of *C*_max_ and AUC_24h_ values between the cohorts should be interpreted with caution. For adafosbuvir, ALS-022399, and ALS-022227, *C*_max_ and AUC_24h_ were generally within the same range between cohorts. For odalasvir, the *C*_max_ and AUC_24h_ values at Week 4 were in the same range in both cohorts. For simeprevir, mean *C*_max_ and AUC_24h_ values at Week 4 were higher in Cohort 2 ([individual values as *n* = 2] *C*_max_: 3540 ng/mL, 9390 ng/mL; AUC_24h_: 53,373 ng.h/mL, 153,138 ng.h/mL) compared with mean values in Cohort 1 (*C*_max_: 2197 ng/mL; AUC_24h_: 26,898 ng.h/mL), and in one of the two patients with cirrhosis (Cohort 2), the exposure was considerably higher.

Graphical evaluation showed no apparent relationship between PR interval (absolute and change from baseline) and odalasvir plasma concentrations (presented in Supplementary Fig. S1).

One patient in Cohort 1 experienced Grade 3 increased AST and Grade 4 increased ALT. In this patient, the plasma pre-dose concentration level of simeprevir at Week 8, when the increase was observed, was the highest value among patients without cirrhosis (2168 ng/mL), although plasma concentrations of adafosbuvir, ALS-022399, ALS-022227, and odalasvir were comparable to those of other patients. Of note, ALT increases were not observed in other patients with similar or higher plasma concentrations of simeprevir.

Of the seven patients who experienced cardiac events, one had the highest pre-dose concentration of odalasvir at Weeks 2, 4, 6, and 8 (474–889 ng/mL) among patients, and a different patient had the maximum *C*_max_ of odalasvir (546 ng/mL) (both Cohort 1), of patients who consented to intensive pharmacokinetic sampling (pharmacokinetic sub-study [10 patients]). For the other five patients, their pre-dose (*C*_trough_) concentrations of odalasvir were within the range of those for patients without cardiac events.

## Discussion

OMEGA-3 aimed to explore the safety and efficacy of 8 and 12 weeks’ treatment with the 3-DAA combination, JNJ-4178, specifically in Japanese patients with or without compensated cirrhosis, and who were infected with HCV GT1 or GT2.

Overall, treatment was well tolerated by patients in both treatment cohorts, with no treatment discontinuations. Most AEs were mild-to-moderate in severity. Three patients in total reported Grade 3 AEs, one of them in combination with a Grade 4 AE. This patient had Grade 3 increased AST and Grade 4 increased ALT that may have been related to the study treatment. There was a slightly higher incidence of AEs in Cohort 2 (81.8%) compared with Cohort 1 (68.2%), but the incidence of treatment-related AEs was similar between the cohorts. Mild variations in LVEF readouts were not considered to be clinically significant.

The overall incidence of AEs during the treatment and follow-up phases in Cohort 1 of the present study (68.2%) was within the range observed following 6 or 8 weeks’ treatment with JNJ-4178 in GT1-, GT2-, GT4-, or GT5-infected patients without cirrhosis in the OMEGA-1 study conducted in Europe, Canada, and Singapore (67.2%–69.2%) [16], and with that observed following 6 or 8 weeks’ treatment in GT1- or GT3-infected patients without cirrhosis in the AL-335–604 study conducted in New Zealand (65–89%) [14].

Several AEs reported in OMEGA-3 have previously been reported for this regimen, including headache, respiratory tract infection, increased ALT and AST, and PR interval prolongation [14, 16]. Accordingly, the cases of first-degree atrioventricular block, that occurred in three patients in this study, were considered at least possibly related to the study drugs. All cases were self-limiting and of no clinical significance.

Treatment with JNJ-4178 was efficacious in the population included in the present study, with an SVR12 rate of 100% in patients with or without compensated cirrhosis, regardless of baseline RASs. With the exception of one patient who was lost to follow-up, all patients achieved SVR24. Mean time to on-treatment virologic response was similar in patients with and without compensated cirrhosis (19.0 and 18.6 days, respectively).

Most patients (~ 70%) in the OMEGA-3 study had HCV GT1, and the high SVR12 rates observed in these patients were consistent with those observed with 6 or 8 weeks’ treatment in GT1-infected patients in the AL-335-604 and OMEGA-1 studies [14, 16]. Although slightly reduced efficacy was previously observed in GT2c-infected patients in OMEGA-1 (SVR12 rates: 75.0–83.3%) [16], all patients in the current study had HCV GT2a or GT2b infection [29]. There were no notable differences in efficacy between GT1- or GT2-infected patients, or between those with or without cirrhosis.

Phase II and III studies assessing simeprevir in combination with pegIFN and ribavirin in HCV GT1-infected treatment-naïve Japanese or Asian patients have demonstrated an SVR12 rate of 89% [30] and SVR24 rates of 77–92% [30, 31]. Common AEs from these studies included increased bilirubin-related AEs, rash, and neutropenia [30, 31]. When compared with these studies, JNJ-4178 in the present study was associated with an increased SVR12 rate (100%) and less-severe AEs, with no incidence of increased bilirubin, including in patients with compensated cirrhosis.

The current standard of care for HCV infection in treatment-naïve patients in Japan includes IFN-free DAA combinations of glecaprevir/pibrentasvir, sofosbuvir/ledipasvir, or elbasvir/grazoprevir as first-line options for HCV GT1, whilst sofosbuvir/ribavirin, glecaprevir/pibrentasvir, or sofosbuvir/ledipasvir are recommended for HCV GT2 [8]. The data from the present study add to the growing body of evidence on the efficacy of DAAs in Japanese patients with HCV infection, including those with more advanced disease. In a retrospective analysis of IFN-free DAAs in a cohort of HCV GT1-infected patients where 18.5% of patients had cirrhosis, 12–24 weeks’ treatment with daclatasvir/asunaprevir, sofosbuvir/ledipasvir, ombitasvir/paritaprevir/ritonavir, or elbasvir/grazoprevir achieved SVR12 rates of 91%, 98%, 98%, and 100%, respectively [32]. In another retrospective study of HCV GT1b-infected DAA treatment-naïve patients in Japan, elbasvir/grazoprevir achieved an SVR12 rate of 97%, and all patients with chronic kidney disease stage 4–5 and advanced fibrosis achieved SVR12 [33]. Furthermore, treatment with glecaprevir/pibrentasvir for 8 or 12 weeks has resulted in SVR12 rates of 99% in HCV GT1- or 2-infected Japanese patients, including DAA-experienced patients and those on hemodialysis [34].

In the present study, intensive pharmacokinetic sampling showed that exposures (*C*_max_ and AUC_24h_) at Week 4 were, overall, within the same range in Cohorts 1 and 2 for adafosbuvir (and its metabolites ALS-022399 and ALS-022227) and odalasvir, but higher in Cohort 2 compared with Cohort 1 for simeprevir. However, these data should be interpreted with caution due to the small number of patients in Cohort 2 with intensive pharmacokinetic data available. AUC_24h_ for adafosbuvir, odalasvir, and simeprevir was much higher in OMEGA-3 than exposures previously observed in non-Japanese patients in the OMEGA-1 study after 6 or 8 weeks’ treatment with JNJ-4178 [16].

For *C*_trough_, data were available for a larger number of patients in Cohorts 1 and 2. The lower odalasvir *C*_trough_ levels in Cohort 2 were consistent with other NS5A inhibitors, where hepatic impairment tends to decrease exposure, whilst higher simeprevir *C*_trough_ levels have previously been described in patients with cirrhosis [35].

A strength of our study was that patients with and without cirrhosis were enrolled, which was reflective of the general population with chronic HCV infection. Limitations of the study included the limited sample size (*N* = 33), that only 11 patients were enrolled in Cohort 2 (and consequently only two participated in the pharmacokinetic sub-study), plus the open-label design. The enrollment of only DAA treatment-naïve patients was a further limitation, as—notably—low rates of SVR12 with another DAA combination (elbasvir/grazoprevir) have been observed in HCV GT1-infected Japanese patients who failed to respond to prior DAAs [33].

As mentioned above, and due to reasons unrelated to safety, the sponsor made a decision to discontinue the JNJ-4178 clinical program along with their HCV development program [23]. However, the results of the OMEGA-3 study, together with the recently completed OMEGA-1 study, make an important contribution to the body of evidence suggesting that with the combination of multiple modes of action, shorter regimens than those currently available could be considered for the treatment of chronic HCV infection.

## Conclusions

In conclusion, the OMEGA-3 study provides further understanding of the efficacy and tolerability of JNJ-4178 as a short-duration therapy in HCV GT1- and GT2-infected patients, with or without cirrhosis. Of note, after 12 weeks’ treatment, SVR12 rates of 100% were achieved in a small number of Japanese patients with compensated cirrhosis, which is typically a difficult-to-treat patient population and one currently with limited treatment options in Japan.

## Electronic supplementary material

Below is the link to the electronic supplementary material.

Supplementary file1 (DOCX 227 kb)

## Data Availability

The data sharing policy of Janssen Pharmaceutical Companies of Johnson & Johnson is available at https://www.janssen.com/clinical-trials/transparency. As noted on this site, requests for access to the study data can be submitted through Yale Open Data Access (YODA) Project site at https://yoda.yale.edu.
