# Supplementary material for: JNJ-4178 (adafosbuvir, odalasvir, and simeprevir) in Japanese patients with chronic hepatitis C virus genotype 1 or 2 infection with or without compensated cirrhosis: the Phase IIa OMEGA-3 study
Source: J Gastroenterol. 2020 Feb 17;55(6):640–52. doi: 10.1007/s00535-020-01672-0 (PMC7242285; doi:10.1007/s00535-020-01672-0)
Supplement: Supplementary file 1 — Supplementary file1 (DOCX 227 kb) [file 535_2020_1672_MOESM1_ESM.docx]

# Electronic supplementary material

**Table S1** Baseline polymorphisms considering the 8 NS3 positions of interest^a^, the
8 NS5A positions of interest^b^, and the 9 NS5B positions of interest^c^ by HCV genotype

| n (%) | | **Cohort 1:  patients with no cirrhosis  (N=22)** | **Cohort 2:  patients with compensated cirrhosis**  **(N=11)** | **Total (N=33)** |
| --- | --- | --- | --- | --- |
| ***NS3*** | |  |  |  |
| *GT1b-infected patients* | |  |  |  |
| Sequencing data available | | 15 | 8 | 23 |
| No NS3 polymorphism at  8 positions of interest | | 3 (20.0) | 1 (12.5) | 4 (17.4) |
| 1 or more NS3 polymorphism at 8 positions of interest | | 12 (80.0) | 7 (87.5) | 19 (82.6) |
| Q80L+S122G+V170I | | 0 | 1 (12.5) | 1 (4.3) |
| Q80L+V170I | | 1 (6.7) | 0 | 1 (4.3) |
| S122G | | 2 (13.3) | 2 (25.0) | 4 (17.4) |
| S122S/N | | 1 (6.7) | 0 | 1 (4.3) |
| S122N+V132I+V170I | | 0 | 1 (12.5) | 1 (4.3) |
| S122S/N+V132I+V170I | | 1 (6.7) | 0 | 1 (4.3) |
| S122T+V132I+V170I | | 0 | 1 (12.5) | 1 (4.3) |
| S122G+V170I | | 1 (6.7) | 1 (12.5) | 2 (8.7) |
| V132I | | 4 (26.7) | 1 (12.5) | 5 (21.7) |
| V170I | | 1 (6.7) | 0 | 1 (4.3) |
| V170V/I | | 1 (6.7) | 0 | 1 (4.3) |
| *GT2-infected patients* | |  |  |  |
| Sequencing data available | | 7 | 3 | 10 |
| No NS3 polymorphism at  8 positions of interest | | 0 | 0 | 0 |
| 1 or more NS3 polymorphism at 8 positions of interest | | 7 (100) | 3 (100) | 10 (100) |
| K122R+I132L^d^ | | 1 (14.3) | 2 (66.7) | 3 (30.0) |
| I132L^d^ | | 6 (85.7) | 1 (33.3) | 7 (70.0) |
| ***NS5A*** | |  |  |  |
| *GT1b-infected patients* | |  |  |  |
| Sequencing data available | | 15 | 8 | 23 |
| No polymorphism at 8 NS5A positions of interest | | 9 (60.0) | 4 (50.0) | 13 (56.5) |
| 1 or more polymorphism at  8 NS5A positions of interest | | 6 (40.0) | 4 (50.0) | 10 (43.5) |
| L28M+R30Q | | 1 (6.7) | 1 (12.5) | 2 (8.7) |
| L28M+R30Q+Y93H | | 0 | 1 (12.5) | 1 (4.3) |
| R30Q | | 1 (6.7) | 0 | 1 (4.3) |
| R30Q+L31M | | 1 (6.7) | 0 | 1 (4.3) |
| L31M | | 1 (6.7) | 0 | 1 (4.3) |
| P58S | | 1 (6.7) | 0 | 1 (4.3) |
| A92A/T | | 0 | 1 (12.5) | 1 (4.3) |
| Y93H | | 0 | 1 (12.5) | 1 (4.3) |
| Y93Y/H | | 1 (6.7) | 0 | 1 (4.3) |
| *GT2-infected patients* | |  |  |  |
| Sequencing data available | | 7 | 3 | 10 |
| No polymorphism at 8 NS5A positions of interest | | 0 | 0 | 0 |
| 1 or more polymorphism at  8 NS5A positions of interest | | 7 (100) | 3 (100) | 10 (100) |
| F28L | | 1 (14.3) | 0 | 1 (10.0) |
| K30K/R+L31M | | 1 (14.3) | 0 | 1 (10.0) |
| L31M | | 5 (71.4) | 3 (100) | 8 (80.0) |
| ***NS5B*** |  | |  |  |
| *GT1b-infected patients* |  | |  |  |
| Sequencing data available | 15 | | 8 | 23 |
| No polymorphism at 9 NS5B positions of interest | 8 (53.3) | | 3 (37.5) | 11 (47.8) |
| 1 or more polymorphism at  9 NS5B positions of interest | 7 (46.7) | | 5 (62.5) | 12 (52.2) |
| N142S+C316N | 1 (6.7) | | 0 | 1 (4.3) |
| C316N | 6 (40.0) | | 5 (62.5) | 11 (47.8) |
| *GT2-infected patients* |  | |  |  |
| Sequencing data available | 7 | | 3 | 10 |
| No polymorphism at 9 NS5B positions of interest | 7 (100) | | 3 (100) | 10 (100) |
| 1 or more polymorphism at  9 NS5B positions of interest | 0 | | 0 | 0 |

^a^The 8 NS3 positions of interest are: 43, 80, 122, 132, 155, 156, 168, and 170.
^b^The 8 NS5A positions of interest are 28, 29, 30, 31, 32, 58, 92, and 93.
^c^The 9 NS5B positions of interest are 96, 142, 159, 223, 226, 282, 316, 320, and 321.
^d^Simeprevir RASs (substitutions with an *in vitro* fold change in 50% effective concentration of simeprevir of >2).

*GT* genotype; *RAS* resistance-associated substitution.

**Table S~~1~~2** Pharmacokinetic parameters for adafosbuvir and its metabolites (ALS-022399 and ALS-022227), odalasvir, and simeprevir at Week 4 (intensive sampling pharmacokinetic sub-study)

| **Study drug or metabolite** | **Pharmacokinetic parameter** | **Cohort 1: patients with no cirrhosis (n=8)** | **Cohort 2: patients with ~~no~~ compensated cirrhosis (n=2)** |
| --- | --- | --- | --- |
| Adafosbuvir | C_max_, ng/mL | 903 (493) | 964; 1430 |
|  | AUC_24h_, ng.h/mL | 1656 (977) | 3022; 3032 |
| ALS-022399 | C_max_, ng/mL | 195 (68.8) | 249; 412 |
|  | AUC_24h_, ng.h/mL | 864 (267) | 1397; 1750 |
| ALS-022227 | C_max_, ng/mL | 974 (554) | 563; 566 |
|  | AUC_24h_, ng.h/mL | 6688 (3116) | 5110; 5054 |
| Odalasvir | C_max_, ng/mL | 342 (113) | 318; 450 |
|  | AUC_24h_, ng.h/mL | 6320 (1898) | 4958; 7177 |
| Simeprevir | C_max_, ng/mL | 2197 (906) | 3540; 9390 |
|  | AUC_24h_, ng.h/mL | 26898 (9678) | 53373; 153138 |

Data are mean (SD) for Cohort 1. Individual values are shown for Cohort 2.
*AUC_24h_* area under the plasma concentration–time curve 0–24 h post-dose, *C_max_* maximum observed analyte concentration, *SD* standard deviation.

**Fig S1.** PR interval (mean of triplicate measurements) change from baseline versus odalasvir predose concentrations after administration of adafosbuvir 800 mg QD, odalasvir 25 mg QD, and simeprevir 75 mg QD


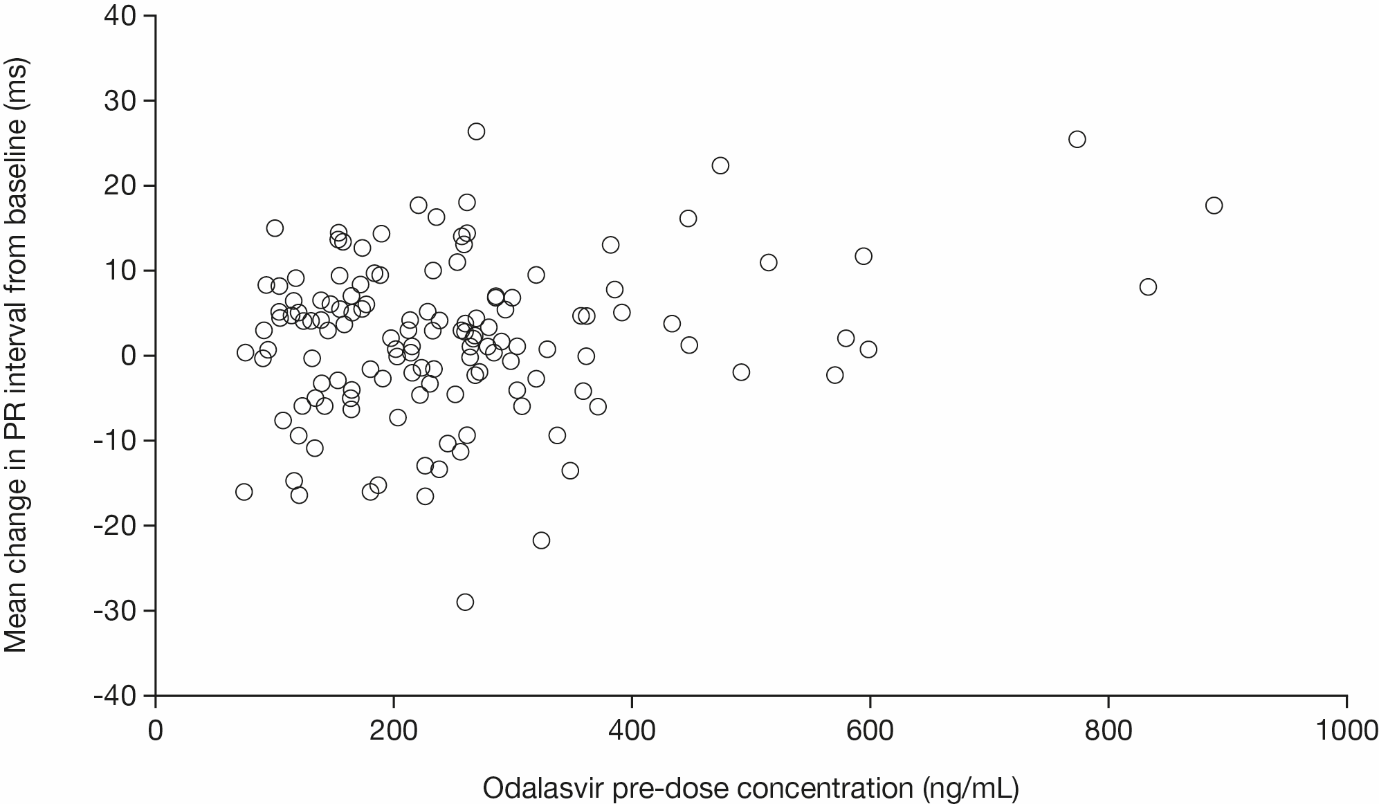


*QD* once daily.
